# Clinical Usability of Exercise Prescription Apps for Professional Use: Systematic Review and Multidimensional Evaluation

**DOI:** 10.2196/77616

**Published:** 2026-03-25

**Authors:** Cheng-Hao Wu, Che-Ning Chang, Chu-Fang Chang, Ming-Hwai Lin, Hsing-Yu Chen, Yu-Chun Chen

**Affiliations:** 1 Department of Family Medicine, Taipei Veterans General Hospital, Taipei, Taiwan; 2 Department of Family Medicine, MacKay Memorial Hospital, Taipei, Taiwan; 3 Center for Traditional Chinese Medicine, Chang Gung Memorial Hospital, Guishan, Taiwan, Taiwan; 4 Taipei Veterans General Hospital, Yuli Branch, No. 91, Xinxing St., Yuli Township, Hualien County, Taiwan, 886 38883141, 886 3883141

**Keywords:** exercise prescription, mHealth apps, behavior change techniques, FITT principle, digital health, app usability, clinical evaluation, mobile health, rehabilitation, exercise adherence

## Abstract

**Background:**

Exercise prescription is a structured and individualized intervention that requires appropriate progression, tailoring, and behavioral support to ensure safety and long-term effectiveness. With the expansion of mobile health technologies, exercise prescription apps are increasingly used to support the remote delivery of prescribed exercise programs. However, the extent to which widely adopted apps align with established clinical standards remains unclear.

**Objective:**

This study aimed to evaluate the clinical usability of popular, no-cost exercise prescription apps from a professional perspective, focusing on clinical integrity, intervention fidelity, behavioral mechanisms, and clinician-assessed digital usability.

**Methods:**

A systematic search of Google Play and the Apple App Store identified widely adopted apps that enable clinician-directed exercise prescription. Eligible apps were evaluated using established frameworks, including the frequency, intensity, time, and type (FITT) and FITT, volume, and progression (FITT-VP) principles; the Consensus on Exercise Reporting Template (CERT); the Behavior Change Technique Taxonomy version 1 (BCTTv1); and the Mobile App Rating Scale (MARS). Descriptive analyses and interrater reliability assessments were performed.

**Results:**

Six apps met the inclusion criteria. All satisfied the basic FITT requirements; however, none incorporated explicit guidance on exercise progression or individualized adjustment consistent with the FITT-VP principles. CERT evaluation demonstrated comprehensive reporting of structural components but a consistent absence of progression logic, tailoring strategies, and adverse event documentation. Although multiple behavior change techniques were identified, several techniques considered important for graded progression and sustained adherence in unsupervised settings were infrequently implemented or absent. Overall app quality was moderate, characterized by strong functionality but limited engagement. Only 2 apps reported evidence of scientific evaluation.

**Conclusions:**

Widely adopted exercise prescription apps meet fundamental structural requirements but do not fully support the progressive and individualized processes central to clinical exercise prescription. These findings highlight a gap between structural prescription delivery and independent clinical exercise management. Exercise prescription apps may therefore be most appropriately positioned as adjunctive tools within clinician-guided or hybrid care models. Future development should prioritize transparent progression mechanisms, individualized adjustment, and the implementation of clinically relevant behavior change strategies to enhance safety and long-term effectiveness.

## Introduction

Exercise prescription is a structured and individualized program of exercise*,* systematically designed to improve health or functional capacity, and these programs are commonly developed by specialists on the basis of patients’ clinical status, goals, and needs [1]. Physicians in physical medicine and rehabilitation, as well as in primary care, routinely prescribe exercise as both treatment and secondary prevention for a broad range of conditions, including musculoskeletal, cardiovascular, neurological, and pulmonary diseases [2-9]. Given this wide scope of application, precision is essential to ensure both safety and effectiveness. According to the American College of Sports Medicine (ACSM), exercise prescription should specify exercise frequency, intensity, time, and type (FITT), with additional consideration of volume and progression (FITT-VP) to support appropriate physiological adaptation and minimize the risk of adverse events [10]. When these elements are appropriately defined and individualized, exercise therapy can deliver evidence-based benefits while reducing the likelihood of exercise-related harm [11].

With the expansion of mobile health (mHealth) technologies, exercise prescription apps have emerged as tools to support the remote delivery of prescribed exercise programs. These systems typically consist of a clinician-facing prescription platform and a patient-facing app, enabling clinicians to design structured exercise programs that patients can access via mobile devices or computers. Prior studies indicate that digitally supported exercise interventions can be feasible, cost-effective, and minimally disruptive to daily life while maintaining patient satisfaction with outpatient services [12-15]. Randomized controlled trials have further demonstrated improvements in adherence to home exercise programs and increased confidence in exercising [16]. Since the COVID-19 pandemic, the growing adoption of digital health solutions has also expanded the use of online training platforms and home-based exercise apps [17-20]. Moreover, app-supported exercise prescription has shown potential benefits in specific clinical populations, including individuals with sarcopenia [21], myocardial infarction [22], or long COVID [23].

Despite growing evidence supporting the feasibility and effectiveness of digitally delivered exercise interventions, concerns remain regarding the professionalism and completeness of exercise prescriptions generated by mobile apps [24]. Compared with face-to-face care, mobile platforms may limit the delivery of detailed instructions, real-time biomechanical feedback, and hands-on correction, all of which are integral to safe and effective exercise prescription. From a professional perspective, the suitability of exercise prescription apps depends on their ability to support clinicians in prescribing, monitoring, and adjusting exercise programs in accordance with established clinical standards. Key considerations include whether exercise prescriptions adequately address essential ACSM components, whether intervention delivery is transparent and reproducible, and whether behavioral strategies are incorporated to support adherence in unsupervised or remote settings. In addition, the rapid development cycles of commercial apps raise concerns regarding usability, consistency, and quality from a clinical perspective [25]. While existing evaluations of health apps often focus on general users, single-disease outcomes, or basic activity tracking [26-30], evidence remains limited regarding the extent to which widely used exercise prescription apps meet professional requirements for clinical prescription and implementation.

To address these gaps, this study adopted a clinician-centered, multidimensional evaluation framework to assess whether popular, no-cost exercise prescription apps are suitable for professional clinical use. The evaluation focused on 4 complementary domains reflecting core requirements of exercise prescription in real-world practice: clinical integrity of exercise prescriptions, intervention fidelity and transparency, behavioral mechanisms supporting adherence, and digital usability and overall app quality. These domains were operationalized using established and validated instruments, including the FITT and FITT-VP principles, the Consensus on Exercise Reporting Template (CERT), the Behavior Change Technique Taxonomy version 1 (BCTTv1), and the Mobile App Rating Scale (MARS). By integrating these perspectives, this study extends beyond general app quality or user-centered evaluations and provides a structured professional assessment of exercise prescription apps intended for clinician-guided care.

## Methods

### Study Design and Conceptual Framework

**Figure 1.**
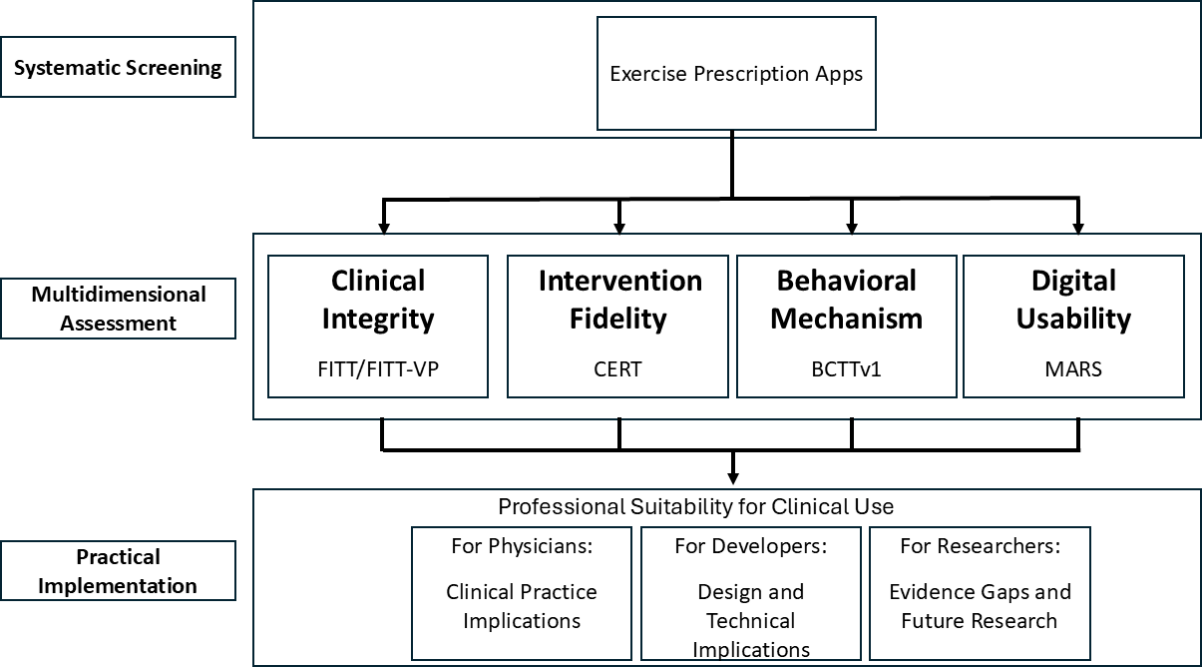
Conceptual framework for clinician-centered evaluation of exercise prescription apps. BCTTv1: Behavior Change Technique Taxonomy version 1; CERT: Consensus on Exercise Reporting Template; FITT: frequency, intensity, time, and type; FITT-VP: frequency, intensity, time, type, volume, and progression; MARS: Mobile App Rating Scale.

This study adopted a clinician-centered, cross-sectional evaluation design to assess exercise prescription apps intended for professional use. A multidimensional evaluation framework was applied to reflect the key components of clinical exercise prescription practice (Figure 1).

The framework included four domains: (1) clinical integrity, (2) intervention fidelity, (3) behavioral mechanism, and (4) digital usability. These domains were operationalized using established instruments: the FITT and FITT-VP principles, CERT, BCTTv1, and MARS.

### Ethical Considerations

This study did not involve human participants, human data, or human biological materials. Therefore, institutional ethics board review and approval were not required.

### App Identification and Search Strategy

This review adhered to the PRISMA (Preferred Reporting Items for Systematic Reviews and Meta-Analyses) protocol, as detailed in the PRISMA 2020 statement (Checklist 1) [31]. A systematic search of Google Play and the Apple App Store was conducted on July 13, 2024, using Taiwanese regional settings. The following search terms were used: “exercise prescription AND doctor,” “exercise prescription AND therapist,” and “exercise prescription AND rehabilitation.” Duplicate apps were removed.

Apps were eligible if they were free, available in English or Chinese, designed for adult users, and not disease specific; did not require additional equipment; and allowed clinicians to prescribe exercise programs. To ensure market relevance, only apps available on both platforms with more than 10,000 downloads were included. A comprehensive and transparent description of this search process is provided in Multimedia Appendix 1.

### Evaluation Framework and Domains

#### Clinical Integrity (FITT and FITT-VP)

Clinical integrity was assessed using the FITT and FITT-VP principles [10]. Apps were considered to meet the FITT criteria if all 4 core components were explicitly specified within the prescribed exercise programs. In addition, adherence to FITT-VP required explicit progression rules or defined mechanisms for adjusting the exercise volume or intensity over time.

#### Intervention Fidelity (CERT)

Intervention fidelity was evaluated using CERT, a 16-item checklist designed to assess the completeness and transparency of exercise interventions [32,33]. The CERT domains include materials, provider qualifications, delivery procedures, setting, dosage, tailoring, and adherence. Apps were assessed using a checklist approach to determine whether each CERT item was addressed. Achievement rates were calculated for individual items. The primary outcome was the achievement rate for individual CERT items.

#### Behavioral Mechanism (BCTTv1)

Behavioral mechanisms were evaluated using BCTTv1, a standardized framework for identifying discrete behavior change techniques (BCTs) within interventions [34]. The taxonomy includes 93 techniques and has been extended in mHealth apps to encompass 102 techniques [35]. Each app was systematically reviewed to identify the presence of BCTs according to BCTTv1 definitions. Achievement rates were calculated as the proportion of apps implementing each technique.

In this study, a predefined subset of BCTs within the BCTTv1 was designated as critical BCTs. These techniques were selected on the basis of prior studies examining strategies associated with exercise initiation, maintenance, and physical activity promotion in digital or remote interventions [36-38]. The selected techniques were grouped into three functional categories: (1) behavior initiation, (2) behavior maintenance, and (3) promotion of physical activity (Multimedia Appendix 2). Achievement rates for this predefined subset were calculated separately.

#### Digital Usability (MARS)

Digital usability and overall app quality were evaluated using MARS, which assesses engagement, functionality, aesthetics, and information quality on a 5-point scale [39,40]. To assess the evidence base component of information quality, supporting scientific literature was identified through database searches in PubMed and Google Scholar, as well as through app descriptions and developer websites. Each item was independently rated by 2 reviewers.

#### Sample Assessment Procedure

All included apps were evaluated using their full range of patient-facing functions. Web-based prescription platforms were not assessed.

Each app was independently reviewed by 2 clinicians (CHW and CNC), both resident physicians with more than 3 years of clinical experience and extensive experience using Android and iOS systems. Prior to evaluation, both reviewers completed formal BCTTv1 training and certification and familiarized themselves with the CERT and MARS protocols. To calibrate scoring interpretations, 5 additional health and fitness apps not included in the final sample were jointly reviewed before data collection.

All apps were installed on an Apple iPhone 8 running iOS version 16.3.1 and a Samsung SM A5360 running Android version 13. Reviewers examined all available features for at least 30 minutes per app. Discrepancies in coding were resolved through discussion, and a third reviewer (YCC) was consulted when consensus could not be reached.

#### Data Extraction and Statistical Analysis

For our study, descriptive data were gathered from Google Play on July 29, 2024, using a Taiwanese account and regional settings. We collected information on app titles, developers, versions, and download numbers directly from the app listings. We chose not to include data from the App Store, as it closely mirrored the information available on Google Play but was less extensive. Ratings and review counts were also excluded from our analysis due to their significant variability across different countries, which complicates comparisons due to the apps’ broad international user base. All statistical analyses were conducted using MedCalc Statistical Software (version 22.0.21; MedCalc Software Ltd). Given the exploratory design, descriptive statistics were primarily employed to present the findings. Interrater reliability was also assessed using Cohen κ or intraclass correlation coefficient (ICC) to determine the level of agreement between the 2 raters [41].

## Results

### App Identification and Selection

**Figure 2.**
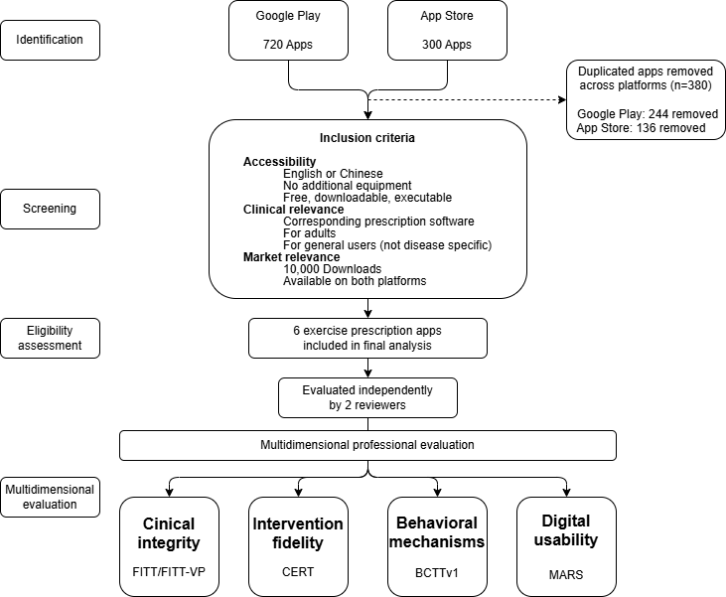
Flow diagram of app identification, screening, eligibility assessment, and multidimensional professional evaluation. BCTTv1: Behavior Change Technique Taxonomy version 1; CERT: Consensus on Exercise Reporting Template; FITT: frequency, intensity, time, and type; FITT-VP: frequency, intensity, time, type, volume, and progression; MARS: Mobile App Rating Scale.

The initial search yielded a total of 1020 apps (each search term yielded 240 apps on Google Play and 100 on the App Store). After removing duplicates and applying the inclusion and exclusion criteria, 6 exercise prescription apps were included in the final analysis (Figure 2). All included apps were available on both platforms; were free to download; supported clinical exercise prescription; and had exceeded 10,000 downloads at the time of assessments.

### App Characteristics

As of July 29, 2024, the 6 apps included in our study demonstrated a wide range of installations, with numbers ranging from 10,000 to 1 million downloads (Table 1). The app developers were based in the United Kingdom, United States, Canada, and Australia. All apps were initially released between 2017 and 2020 and had all been updated in 2023 or 2024.

**Table 1.** Characteristics of included exercise prescription apps (N=6; July 29, 2024).

App characteristic	PhysiApp	MedBridge GO for Patients	Wibbi	TrackActive Pro - Patient App	Rehab Guru Client	Telehab
Number of installs	>1 million	>1 million	>100,000	>10,000	>10,000	>10,000
Developer	Physitrack PLC	MedBridge	Wibbi	Active Health Tech Pty Ltd	Rehab Guru Team	VALD
Country of developer	United Kingdom	United States	Canada	Australia	United Kingdom	Australia
Date of release	Oct 30, 2018	May 15, 2017	Feb 20, 2017	Feb 17, 2017	Aug 1, 2018	May 26, 2020
Version	4.20.0	4.6.3	1.3.1	1.6.7	3.1.0	1.3.0
Date of update	Jul 22, 2024	Apr 3, 2024	Jul 16, 2024	Feb 13, 2024	Oct 26, 2023	Mar 3, 2023

### Clinical Integrity Assessment Based on FITT and FITT-VP Principles

All 6 apps met the FITT criteria, as they explicitly specified the exercise frequency, intensity, time, and type within prescribed exercise programs (Table 2). In contrast, none of the apps met the FITT-VP criteria. Across all apps, explicit guidance on progression of exercise volume or intensity over time was absent, and no app provided decision rules for adjusting exercise prescriptions based on user performance or program duration.

**Table 2.** Multidimensional evaluation results of included exercise prescription apps (N=6; July 29, 2024).

	PhysiApp	MedBridge GO for Patients	Wibbi	TrackActive Pro - Patient App	Rehab Guru Client	Telehab
Clinical integrity						
FITT^a^	Yes	Yes	Yes	Yes	Yes	Yes
FITT-VP^b^	No	No	No	No	No	No
Intervention fidelity						
CERT^c^	10	10	10	8	9	10
Behavioral mechanism						
BCTTv1^d^	18	16	15	14	15	19
Digital usability						
MARS^e^	3.82	3.85	3.36	3.36	3.55	3.99

a FITT: Frequency, intensity, type, time. “Yes” denotes full adherence to all 4 elements.

b FITT-VP: Frequency, intensity, type, time, volume, progression. “Yes” denotes full adherence to all 6 elements.

c CERT: Consensus on Exercise Reporting Template. Score indicates number of checklist items met (maximum=16).

d BCTTv1: Behavior Change Technique Taxonomy version 1. Number of identified behavior change techniques.

e MARS: Mobile App Rating Scale. Mean overall score on a 5-point scale.

### Intervention Fidelity Assessment Based on CERT

**Figure 3.**
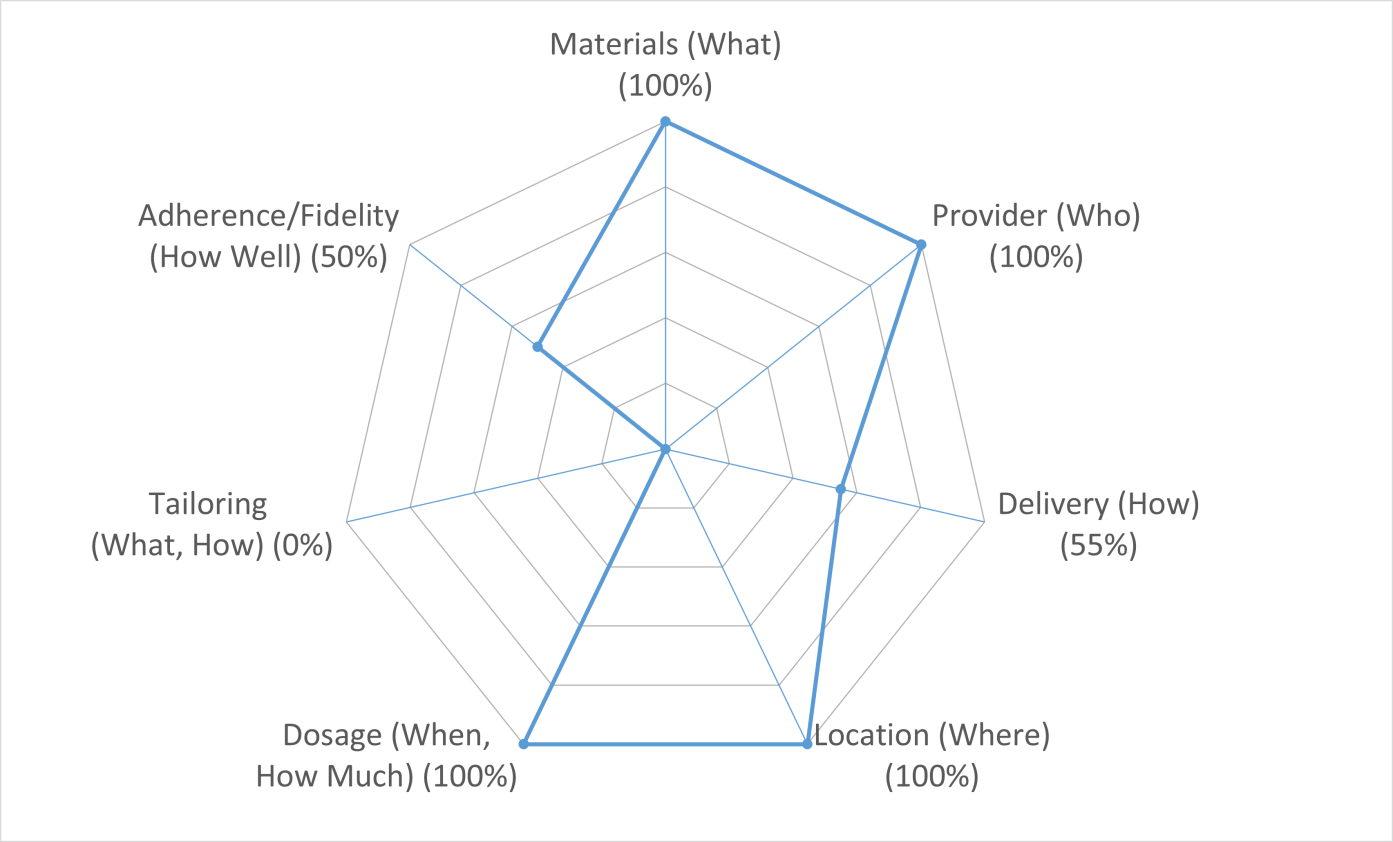
Domain-level reporting patterns of the Consensus on Exercise Reporting Template across exercise prescription apps (N=6; 2024).

Intervention fidelity varied across CERT domains (Table 3 and Figure 3). All 6 apps (100%) achieved complete reporting for core structural elements, including materials, provider qualifications, exercise setting, and dosage. Within the delivery domain, all apps specified whether exercises were performed individually or in groups and whether sessions were supervised or unsupervised. Of the 6 apps, 5 apps (83%) reported methods for assessing exercise adherence, and 4 apps (67%) included nonexercise components such as educational or dietary content.

**Table 3.** Item-level achievement rates for the Consensus on Exercise Reporting Template (CERT) across included exercise prescription apps (N=6; 2024).

Abbreviated item description of CERT	Achievement rate
Materials (what)	
Detailed description of the type of exercise equipment	100%
Provider (who)	
Description of qualifications/expertise/training of instructor	100%
Delivery (how)	
Description of whether exercises are performed individually or in a group	100%
Description of whether exercises are supervised/unsupervised	100%
Description of the measurement/reporting of adherence to exercise	83%
Detailed description of motivation strategies	0%
Detailed description of the decision rule(s) of exercise progression	0%
Detailed description of how the exercise program was progressed	0%
Description of each exercise to enable replication (eg, illustrations and photographs)	0%
Description of any home programmed component	100%
Description of any nonexercise component	67%
Description of the type/number of adverse events that occurred during exercise	0%
Location (where)	
Description of exercise setting	100%
Dosage (when and how much)	
Description of exercise intervention and dosage	100%
Tailoring (what and how)	
Description of whether exercises are generic or tailored to the individual	0%
Detailed description of how exercises are tailored to the individual	0%
Decision rule for starting level of exercise	0%
Adherence/fidelity (how well)	
Description of how adherence or fidelity to the exercise intervention is assessed/measured	100%
Description of the extent to which the intervention was delivered as planned	0%

In contrast, no app (0%) reported motivational strategies, decision rules for exercise progression, or descriptions of how exercise programs were progressed over time. Similarly, none of the apps described whether or how exercises were tailored to individual users or how starting exercise levels were determined. Reporting of adverse events was absent across all apps, and none documented whether the prescribed intervention was delivered as planned. The comprehensive table with respective scoring can be found in Multimedia Appendix 3. Interrater reliability for CERT assessment was high (Cohen κ=0.88).

### Behavioral Mechanism Assessment Based on BCTTv1

#### Overall Implementation of BCTs

Across the 6 included apps, a limited but heterogeneous set of BCTs was identified. When assessed against the original 93-item BCTTv1 taxonomy, the mean number of techniques implemented by apps was 13.2 (SD 1.77; range 11‐16). When extended mHealth categories were included (102 techniques), the mean number of techniques increased to 16.2 (SD 1.77; range 14‐19). The interrater reliability for BCT coding was high (Cohen κ=0.85).

Fourteen techniques were implemented across all apps (100% achievement), including goal setting (behavior), action planning, feedback on behavior, self-monitoring of behavior, instruction on how to perform the behavior, demonstration of the behavior, and behavioral practice or rehearsal. Additional techniques were implemented in a subset of apps. The full list of identified BCTs and their achievement rates is presented in Table 4, and detailed coding results, including the extended 102-technique classification, are provided in Multimedia Appendix 4.

**Table 4.** Achievement rates of behavior change techniques (BCTs) identified from the full BCTTv1^a^ taxonomy across included exercise prescription apps (N=6; 2024), ordered by frequency of implementation.

BCTTv1 code	BCT	Critical BCT^b^?	Achievement rate
1.1	Goal setting (behavior)	Yes	100%
1.4	Action planning	Yes	100%
1.5	Review behavior goal(s)	No	100%
2.1	Monitoring of behavior by others without feedback	No	100%
2.2	Feedback on behavior	Yes	100%
2.3	Self-monitoring of behavior	Yes	100%
4.1	Instruction on how to perform a behavior	Yes	100%
6.1	Demonstration of the behavior	Yes	100%
8.1	Behavioral practice/rehearsal	Yes	100%
9.1	Credible source	No	100%
12.6	Body changes	No	100%
17.1	Tailoring to demographic characteristics	No	100%
17.2	Tailoring to health status	No	100%
17.4	Adjusting intervention content to performance	No	100%
5.1	Information about health consequences	No	66%
10.4	Social reward	No	50%
1.3	Goal setting (outcome)	Yes	33%
1.7	Review outcome goal(s)	No	33%
2.5	Monitoring outcome(s) of behavior by others without feedback	No	33%

a BCTTv1: The Behavior Change Technique Taxonomy version 1. It comprises 102 distinct techniques. Only techniques identified in at least 1 app are presented; all remaining BCTTv1 items had an achievement rate of 0% across the evaluated exercise prescription apps.

b Critical BCTs were defined a priori as techniques with established relevance to exercise initiation, long-term maintenance, or physical activity promotion in digital or remote intervention contexts.

#### Predefined Clinically Relevant BCTs

Table 5 shows the predefined critical BCTs grouped into 3 functional categories: behavior initiation, behavior maintenance, and promotion of physical activity. Within behavior initiation, demonstration of the behavior and behavioral practice or rehearsal were incorporated by all apps (6/6, 100%), whereas biofeedback and graded tasks were not identified (0/6, 0%). For behavior maintenance, action planning and instruction on how to perform the behavior were universally implemented (6/6, 100%), while prompts or cues, graded tasks, and self-reward were absent (0/6, 0%). Among techniques associated with physical activity promotion, goal setting (behavior), action planning, feedback on behavior, and self-monitoring were consistently implemented, whereas social support, restructuring the physical environment, and framing or reframing were not identified.

**Table 5.** Achievement rates of predefined critical behavior change techniques across functional roles among included exercise prescription apps (N=6; 2024).

Functional category and behavior change techniques (BCTTv1^a^ code)	Achievement rate
Behavior activation	
6.1 Demonstration of the behavior	100%
8.1 Behavioral practice/rehearsal	100%
2.6 Biofeedback	0%
8.7 Graded tasks	0%
Behavior maintenance	
1.4 Action planning	100%
4.1 Instruction on how to perform a behavior	100%
8.1 Behavioral practice/rehearsal	100%
7.1 Prompts/cues	0%
8.7 Graded tasks	0%
10.9 Self-reward	0%
Physical activity promotion	
1.1, 1.3 Goal setting (behavior and outcome)	100%
1.4 Action planning	100%
2.2 Feedback on behavior	100%
2.3, 2.4 Self-monitoring of behavior with its outcome(s)	100%
10.1‐10.11 Items in group “Reward and threat”	50%
3.1‐3.3 Items in group “Social support”	0%
12.1 Restructuring the physical environment	0%
13.2 Framing/reframing	0%

a BCTTv1: The Behavior Change Technique Taxonomy version 1.

### Digital Usability Based on MARS

**Figure 4.**
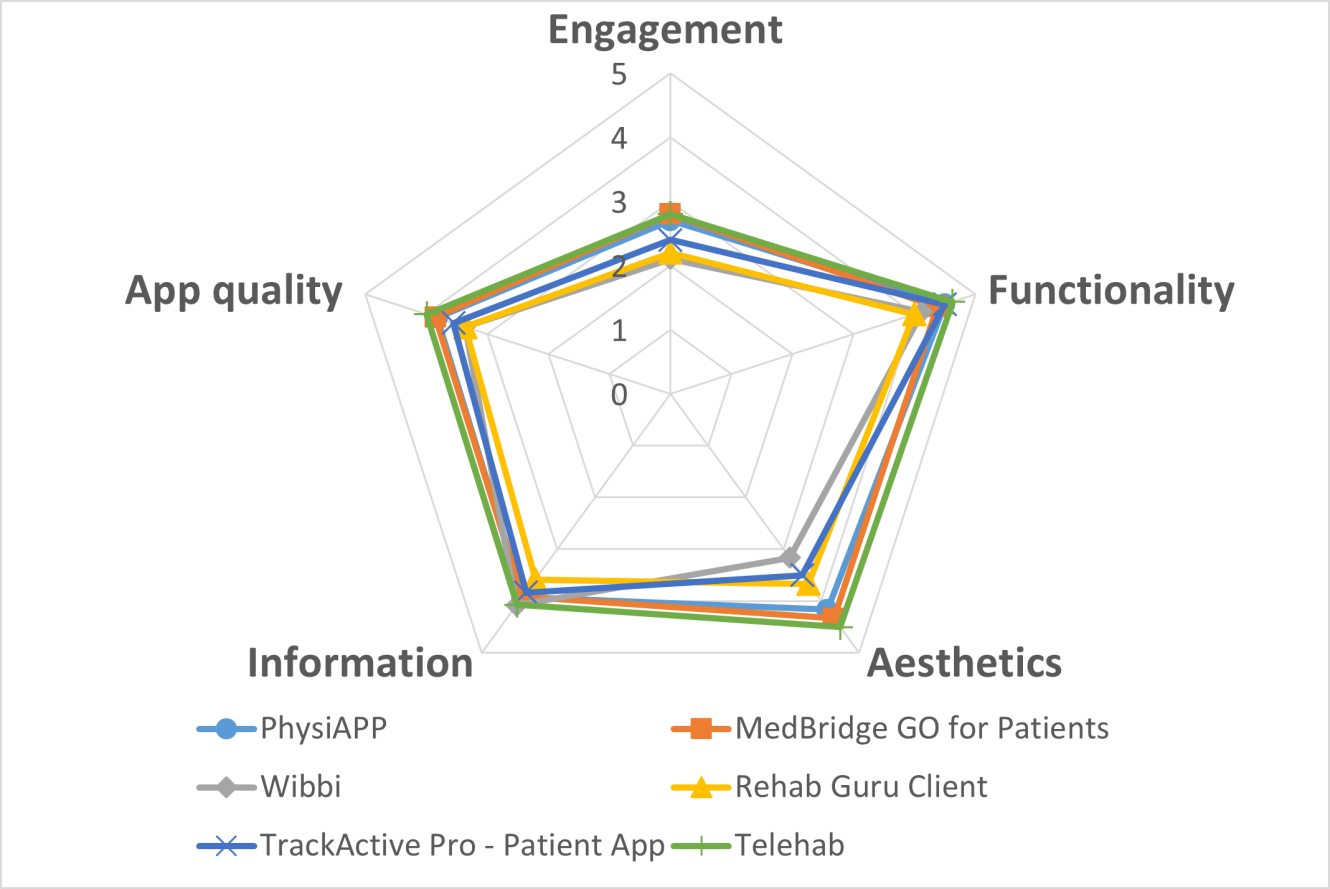
Radar chart illustrating the Mobile App Rating Scale scores of the 6 included exercise prescription apps (N=6; 2024).

Assessment of overall app quality using MARS yielded a mean score of 3.66 (SD 0.25) across the 6 apps, with individual app scores ranging from 3.36 to 4.00 (Table 6 and Figure 4). Among MARS domains, engagement received the lowest ratings, with a mean score of 2.50 (SD 0.28; range 2.10‐2.80), indicating limited use of interactive, motivational, or personalized features. In contrast, functionality was rated highest, achieving a mean score of 4.36 (SD 0.22; range 4.00‐4.63), reflecting stable performance, ease of navigation, and technical reliability across all apps. Aesthetic quality was rated moderately to highly, with a mean score of 3.89 (SD 0.48; range 3.17‐4.50), and information quality was rated similarly, with a mean score of 3.90 (SD 0.18; range 3.58‐4.07). Comprehensive tables with both reviewer and respective scoring results are provided in MultimediaAppendices 5  6. The interrater reliability of MARS was assessed using the ICC (2-way random effects, absolute agreement, average measures). The resulting ICC was 0.71 (95% CI 0.59‐0.81), indicating good reliability.

**Table 6.** Digital usability and quality scores of included exercise prescription apps (N=6; 2024) assessed using the Mobile App Rating Scale (MARS).

MARS domain	PhysiApp	MedBridge GO for Patients	Wibbi	TrackActive Pro - Patient App	Rehab Guru Client	Telehab	MARS score, mean (SD)^a^
Engagement	2.70	2.80	2.10	2.20	2.40	2.80	2.50 (0.28)
Functionality	4.50	4.38	4.13	4.00	4.50	4.63	4.36 (0.22)
Aesthetics	4.17	4.33	3.17	3.67	3.50	4.50	3.89 (0.48)
Information	3.93	3.92	4.08	3.58	3.83	4.07	3.90 (0.17)
Overall quality	3.82	3.86	3.37	3.36	3.56	4.00	3.66 (0.25)

a The maximum possible MARS score is 5.0, based on a 5-point Likert scale ranging from 1 (inadequate) to 5 (excellent).

Our review of the evidence item revealed that by July 2024, only 2 apps among the cohort had undergone scientific evaluation, suggesting a lack of empirically validated health apps in the current market.

## Discussion

### Principal Results

This study uses an integrated, clinician-centered, multidimensional evaluation framework to provide a novel professional perspective on how exercise prescription apps operate in real-world clinical contexts. Overall, popular, no-cost exercise prescription apps were found to meet the basic structural requirements for exercise prescription but fell short of key standards required for independent clinical use. Although prescriptions consistently specified core FITT elements, the absence of explicit progression and individualized adjustment limited alignment with established clinical practice. While multiple BCTs were identified, those critical for sustaining exercise behavior in unsupervised or remote settings were frequently lacking. Despite strong technical functionality and high information quality, low engagement scores suggest that usability alone is insufficient to support long-term adherence.

When considered collectively across domains, these apps nonetheless demonstrate operational strengths, including structured translation of prescriptions into actionable tasks, continuous visibility of adherence, low cognitive load instruction delivery, and delegation of routine monitoring. Taken together, these findings highlight a gap between clinical expectations for exercise prescription and the current capabilities of widely used digital tools, supporting the role of exercise prescription apps as adjuncts within clinician-guided care rather than as stand-alone prescribing solutions.

### Conceptual Foundation and Innovation of the Multidimensional Evaluation Framework

This study introduces a clinician-centered evaluation framework that integrates clinical exercise prescription standards, behavior change theory, and digital usability into a unified, practice-oriented model. Unlike prior evaluations of physical activity and mHealth apps that have primarily examined usability, engagement, or behavior change features in isolation [26-28], this framework situates app assessment within the logic of real-world clinical exercise prescription and decision-making. By aligning established instruments with sequential stages of professional practice, including prescription structure and progression logic (FITT and FITT-VP), transparency and reproducibility of intervention delivery (CERT), support for exercise initiation and maintenance (BCTTv1), and interface quality and engagement (MARS), the framework enables clinicians to directly compare app features with clinical expectations. In doing so, it provides a shared reference to guide both informed clinical selection and future app development.

### Adherence to FITT Principles and the Need for Enhanced Program Progression in Exercise Apps

Unlike general physical activity apps that often fail to meet the basic FITT criteria [24,42], the specialized apps in our study successfully incorporated these fundamental elements. However, the primary clinical limitation lies in their failure to satisfy the full FITT-VP framework, specifically regarding structured progression and individualized adjustment. In established exercise prescription practice, progression is essential for achieving physiological adaptation while minimizing injury risk, particularly by avoiding abrupt increases in exercise volume or intensity [43,44]. Clear guidance on how and when to advance exercises is also critical for informed consent and may facilitate patient engagement and adherence.

In addition, limited support for tailoring exercise programs to individual needs constrains the safe use of these tools in populations such as older adults and individuals with chronic diseases, for whom inappropriate exercise loading may increase the risk of adverse events [45]. The absence of explicit information regarding potential exercise-related risks further complicates informed decision-making by both clinicians and patients. To optimize the integration of exercise prescription apps into clinical practice, more comprehensive and transparent intervention descriptions are required to support individualized care and the effective translation of evidence-based exercise prescription into real-world settings.

### Intervention Fidelity and Clinical Transparency in App-Based Exercise Prescription

The evaluation of intervention fidelity via the CERT domains revealed a stark disparity between the reporting of static structural elements and dynamic clinical processes. While all apps achieved 100% compliance in describing core components such as materials, provider qualifications, and settings, there was a universal failure (0%) to report motivational strategies, progression logic, or tailoring mechanisms. This deficiency mirrors findings from previous systematic reviews in pregnancy [46] and musculoskeletal rehabilitation [47], which similarly identified a pervasive lack of customization features in mHealth tools. Crucially, the absence of documented decision rules for exercise progression limits the transparency of the intervention. As highlighted by Hansford et al [48] and Conrado Ignacio et al [49], detailed reporting of intervention components—beyond simple dosage parameters—is essential for ensuring reproducibility and clearly defining the functional mechanisms of the prescribed therapy. Consequently, while digital platforms successfully deliver standardized content, the lack of explicit reporting on how exercises are adjusted obscures the clinical reasoning necessary to ensure safety and effectiveness throughout the rehabilitative trajectory.

Furthermore, the total lack of information regarding baseline determination and individual tailoring (0%) highlights a fundamental challenge in digital health. According to updated preparticipation health screening recommendations [50], a comprehensive evaluation of a patient’s physical status and functional capacity is the indispensable prerequisite for ensuring safety before initiating any exercise program. The inability of current apps to document how starting levels are calibrated or adjusted to individual constraints suggests a reliance on generic protocols rather than precise clinical titration. Coupled with the complete omission of adverse event reporting, these findings indicate that current apps function as digital exercise libraries rather than sophisticated therapeutic tools, falling short of the safety and individualization standards required for clinical populations.

### Enhanced BCTs With Challenges in Addressing Supervision and Adherence in Exercise Apps

The exercise prescription apps reviewed in this study incorporated a higher number of BCTs than reported in previous app evaluations [29,30]; the specific selection of strategies remains highly consistent with the broader mHealth literature [29,51-54]. The predominance of self-monitoring, feedback, goal setting, and action planning in our sample mirrors the most frequently identified features in recent content analyses, confirming that these specialized tools adhere to the standard design patterns observed in general physical activity interventions. However, consistent with established behavior change theory, the mere presence of multiple techniques does not ensure effective behavioral support if their selection and configuration are not aligned with the clinical requirements of long-term exercise adherence [55].

From a clinical perspective, several predefined critical BCTs for sustaining exercise behavior in unsupervised or remote settings were absent or infrequently implemented. These included graded tasks, biofeedback, prompts or cues, and self-reward—mechanisms closely associated with progression, self-regulation, and adaptive feedback over time. Their limited representation suggests that current exercise prescription apps are primarily optimized for exercise initiation and short-term compliance rather than for dynamic adjustment and sustained behavior change, which are central to clinical exercise prescription.

Conversely, several frequently implemented techniques that were not classified as critical were consistently present across all apps, including presentation of a credible source, routine review of behavioral goals, monitoring without feedback, and repeated behavioral practice. Although these techniques may have limited independent effects on long-term adherence, they may provide complementary clinical value by reinforcing professional authority, standardizing instruction, and supporting structured follow-up [56,57]. Taken together, these findings indicate that while current exercise prescription apps insufficiently implement several clinically critical BCTs, their operational strengths may still augment clinician-guided or hybrid care models, although they may not effectively function as stand-alone behavioral interventions.

### Digital Usability in Exercise Apps

In our analysis, the exercise prescription apps achieved an average MARS quality score of 3.66 (SD 0.25), consistent with prior studies by Paganini et al [58] and Simões et al [59], which reported moderate overall quality scores for physical activity apps. Paganini et al [58] found the average quality score to be 3.60, with variations across different subcategories: information averaged 3.24; engagement, 3.19; aesthetics, 3.65; and functionality, the highest at 4.35. Simões et al [59] reported a slightly higher overall MARS score of 3.88, with functionality again scoring the highest at 4.30, followed by aesthetics, information quality, and engagement.

Notably, in our study, the score for information quality was higher at 3.90, whereas the engagement score was considerably lower at 2.50. This discrepancy may reflect the more specific and task-oriented nature of our evaluated apps compared to the broader range of apps included in other studies, which might engage users differently. Despite substantial download figures, the apps we reviewed showed limited evidence of effectiveness, a finding that corroborates previous evaluations [60-62], which consistently highlighted a shortage of evidence-based content and professional guideline adherence in the mHealth landscape.

### Clinical Implication

From a clinical perspective, the findings of this study indicate that currently available exercise prescription apps should not be used as stand-alone prescribing tools but rather incorporated into clinician-guided or hybrid care models. Although these apps consistently deliver structured exercise instructions and provide visibility of patient adherence, the absence of explicit progression logic, individualized adjustment, and several clinically critical BCTs limits their capacity to independently support safe and effective exercise prescription. Consequently, clinicians remain essential for determining appropriate starting levels, explaining progression criteria, and managing exercise-related risks, particularly in older adults and individuals with chronic diseases [63].

At the same time, the operational features that are consistently implemented across apps such as standardized exercise demonstrations, routine goal review, adherence tracking, and low cognitive load instruction delivery may still offer complementary clinical value. When used alongside professional judgment, these features can reduce instructional burden, support treatment consistency, and facilitate structured follow-up between clinical encounters. In this context, exercise prescription apps may function as digital extensions of clinician-led care rather than autonomous therapeutic systems.

For developers, these findings underscore the need to move beyond technically feasible features toward closer alignment with clinical exercise prescription standards. Enhancing transparency around progression rules, incorporating mechanisms for individualized adjustment, and prioritizing BCTs associated with long-term adherence may improve the clinical relevance of future applications. Most importantly, adopting a co-design approach—actively involving both clinical practitioners and end users in the development process—is essential to bridge the gap between technical implementation and real-world clinical utility [64,65].

### Limitations

This study has several limitations. First, search results were inherently transient and geographically specific due to localized app store algorithms and the rapidly evolving mobile market [66]. While our semantic search strategy targeted popular, clinically relevant apps rather than an exhaustive catalog, the findings represent a 2024 snapshot, and the currency of these results may diminish, as apps undergo frequent updates. Second, while the interrater reliability was high for CERT and BCT assessments, the MARS evaluation yielded relatively lower consistency, reflecting the subjective nature of aesthetic and engagement metrics; however, all discrepancies were resolved through rigorous consensus-based discussion to ensure data validity.

Third, the CERT and BCTTv1 frameworks, originally designed for research interventions, may not fully capture modern app functionalities such as automated feedback, potentially creating a structural mismatch in scoring. Fourth, we identified the presence of BCTs but did not evaluate the quality of their implementation or their actual impact on user behavior. Furthermore, the lack of longitudinal adherence and clinical outcome data limits our assessment of real-world therapeutic impact.

### Future Direction

While our study establishes fundamental professional quality and usability benchmarks for exercise prescription apps, it serves primarily as a foundational step mapping the current landscape. Future work should build upon these findings by transitioning from quality assessment to rigorous clinical verification.

Specifically, longitudinal randomized controlled trials are needed to determine if the high-scoring, professionally vetted apps identified in this study translate to better patient adherence and health outcomes compared to traditional methods. Furthermore, considering that the effectiveness of digital exercise prescription systems may vary significantly across conditions, these investigations should target specific clinical cohorts—such as patients with cardiac conditions, neurological disorders (eg, poststroke rehabilitation), or musculoskeletal issues (eg, sarcopenia)—rather than general users.

Crucially, to match the complexity of clinical needs, efficacy trials must move beyond self-reported measures and incorporate objective physiological outcomes, such as laboratory data (eg, inflammatory markers and glycemic control) and body composition metrics assessed via bioelectrical impedance analysis. Finally, integrating wearable sensors for real-time biofeedback represents a promising frontier for ensuring that these digital prescriptions are delivered safely and effectively.

### Conclusions

Using a clinician-centered, multidimensional evaluation framework, this study systematically assessed the clinical usability of popular, no-cost exercise prescription apps. Although these apps consistently met basic structural requirements and demonstrated strong technical functionality, they lacked essential features related to progression, individualization, intervention transparency, and sustained behavior change support. As a result, their capacity to independently deliver clinically appropriate exercise prescriptions remains limited.

Nevertheless, the standardized delivery of exercise instructions, structured translation of prescriptions into actionable tasks, and support for adherence monitoring suggest that these apps may still add value within clinician-guided or hybrid care models. Future development should prioritize a co-design approach involving both clinicians and users to bridge existing gaps. This collaborative strategy is essential to align apps with clinical standards and behavior change principles, thereby enhancing their safety, effectiveness, and clinical relevance.

## Supplementary material

10.2196/77616 Multimedia Appendix 1 Detailed search strategy and selection protocol.

10.2196/77616 Multimedia Appendix 2 The behavior change techniques in prior studies coded in the Behavior Change Technique Taxonomy version 1 framework.

10.2196/77616 Multimedia Appendix 3 The Consensus on Exercise Reporting Template checklist with detailed description across included exercise prescription apps (N=6; 2024).

10.2196/77616 Multimedia Appendix 4 The behavior change techniques coded in Behavior Change Technique Taxonomy version 1 framework across included exercise prescription apps (N=6; 2024).

10.2196/77616 Multimedia Appendix 5 The Mobile App Rating Scale scores of included exercise prescription apps on the Android system (N=6; 2024).

10.2196/77616 Multimedia Appendix 6 The Mobile App Rating Scale scores of included exercise prescription apps on the iOS system (N=6; 2024).

10.2196/77616 Checklist 1 PRISMA 2020 checklist.

## Abbreviations

ACSM: American College of Sports Medicine
BCT: behavior change technique
BCTTv1: Behavior Change Technique Taxonomy version 1
CERT: Consensus on Exercise Reporting Template
FITT: frequency, intensity, time, and type
FITT-VP: frequency, intensity, time, type, volume, and progression
ICC: intraclass correlation coefficient
MARS: Mobile App Rating Scale
mHealth: mobile health
PRISMA: Preferred Reporting Items for Systematic Reviews and Meta-Analyses
