# Supplementary material for: Clinical Usability of Exercise Prescription Apps for Professional Use: Systematic Review and Multidimensional Evaluation
Source: JMIR Mhealth Uhealth. 2026 Mar 25;14:e77616. doi: 10.2196/77616 (PMC13015917; doi:10.2196/77616)
Supplement: Multimedia Appendix 1 [file mhealth-v14-e77616-s001.docx]

**Appendix 1: Detailed Search Strategy and Selection Protocol**

**1. Search Strategy and Data Sources**

- **Search Configuration and Timing**

The systematic search was conducted on **July 13, 2024**. To standardize the search environment, the regional settings for both Google Play and the Apple App Store were configured to **Taiwan**. Crucially, to mitigate potential algorithmic bias ("filter bubbles") arising from historical usage data, **dedicated new user accounts were registered on both platforms specifically for this study**. This precautionary measure was implemented to ensure the search results were neutral and not "contaminated" by the researchers' personal download history or preferences.

- **Search Execution**

Following this configuration, a systematic search was performed on both platforms. To ensure a comprehensive identification of apps specifically designed for professional exercise prescription, we employed a composite keyword strategy linking the core intervention with professional roles. The specific search terms used were:

- *“exercise prescription AND doctor”*
- *“exercise prescription AND therapist”*
- *“exercise prescription AND rehabilitation”*
- **Platform Constraints:** The number of retrieved applications was determined by the **inherent display limits** of each platform's search algorithm, rather than an arbitrary cutoff selected by the researchers.
- **Google Play:** Restricts the maximum viewable results to approximately **240** per keyword query.
- **App Store:** Limits the display to **100** per keyword query.

Consequently, the initial search yielded a total raw pool of **1,020** potential entries: 720 from Google Play (240 × 3 keywords) and 300 from the App Store (100 × 3 keywords). These raw results were compiled into a master list, and duplicate entries were removed prior to screening.

**2. Inclusion and Exclusion Criteria**

To ensure the identified applications were viable for widespread clinical use and professional auditing, a multi-stage screening process was applied based on the following criteria:

**2.1. Metadata Screening Criteria (Stage 1)** Before downloading, apps were screened based on store metadata (title, description, and basic statistics). Inclusion criteria were:

- **Accessibility:** Free to download and use (freemium models were accepted if core prescription features were free).
- **Language:** Available in English or Chinese.
- **Popularity:** Total downloads exceeding **10,000**, serving as a proxy for a validated and active user base.
- **Cross-Platform Availability:** Available on *both* Google Play and the App Store to ensure universality for diverse patient populations.

Apps were excluded at this stage if their titles or descriptions clearly indicated irrelevance to the research question, such as medication trackers (drug prescriptions), simple pedometers, or general fitness logs lacking a provider interface.

**2.2. Functional Inclusion Criteria (Stage 2)** Apps passing the metadata screen underwent in-depth functional verification. To be included, an app must demonstrate a **"Professional Prescription Ecosystem,"** defined by the following characteristics:

- **Provider-Patient Interface:** The system must consist of a dual interface—a software platform for experts to design/prescribe exercise programs, and a mobile app for patients to receive and view the plan.
- **Standalone Functionality:** The app must operate independently without requiring proprietary external hardware or specific wearable devices.
- **Target Population:** Designed for general adult use rather than pediatric populations.
- **Generalizability:** Applicable to a broad range of conditions rather than being restricted to a single specific disease.

**3. Selection Procedure and Quality Assurance**

To ensure the reliability of the selection process, the screening and verification were conducted by **two independent researchers**. To address the cross-platform requirement efficiently, one researcher primarily assessed the applications on the **Android platform**, while the other assessed the **iOS platform**. Both researchers cross-verified that candidate apps were available and functionally identical on both platforms.

**3.1. In-Depth Verification and Exclusion Examples** During the download and testing phase, the researchers rigorously examined the apps against the functional criteria. Apps were excluded if they failed to support an active prescription model. Specific examples of exclusion include:

- **Disease-Specific Restrictions:** Apps that offered comprehensive prescription tools but were strictly limited to specific cohorts (e.g., an app designed exclusively for **cardiac rehabilitation** patients) were excluded to maintain the study's focus on generalizability.
- **Passive Information Delivery:** Apps that functioned primarily as content aggregators—delivering health news or general exercise education without a mechanism for a professional to actively "prescribe" a specific regimen—were deemed ineligible.

**3.2. Consensus and Adjudication** Any discrepancies regarding app eligibility (e.g., one researcher advocating for inclusion while the other suggested exclusion based on feature interpretation) were first addressed through a consensus-based discussion between the two primary reviewers. In cases where a consensus could not be reached, a **third senior author** was consulted to review the app against the criteria and provide the final adjudication.

**4. Final Selection**

Following this rigorous filtration process, a total of **6 applications** met all inclusion criteria and were included in the final analysis.
