# Supplementary material for: Clinical Usability of Exercise Prescription Apps for Professional Use: Systematic Review and Multidimensional Evaluation
Source: JMIR Mhealth Uhealth. 2026 Mar 25;14:e77616. doi: 10.2196/77616 (PMC13015917; doi:10.2196/77616)
Supplement: Multimedia Appendix 2 [file mhealth-v14-e77616-s002.docx]

| Appendix 2: The behavior change technique(BCT) in prior study coded in Behavior Change Technique Taxonomy version 1 (BCTTv1)framework | |
| --- | --- |
| Items in previous study | Items recoded in BCTT V1 |
|  |  |
| **Items for activating behavior change** | |
| Biofeedback | 2.6 Biofeedback |
| Demonstration of the behavior | 6.1 Demonstration of the behavior |
| Behavioral practice/ rehearsal | 8.1 Behavioral practice/ rehearsal |
| Graded tasks | 8.7 Graded tasks |
| **Items for behavior change maintenance** | |
| Action planning | 1.4 Action planning |
| Instruction on how to perform a behavior | 4.1 Instruction on how to perform a behavior |
| Prompts/cues | 7.1 Prompts/cues |
| Behavioral practice/ rehearsal | 8.1 Behavioral practice/ rehearsal |
| Graded tasks | 8.7 Graded tasks |
| Self-reward | 10.9 Self-reward |
| **Items utilized in fitness technology for increasing physical activity** | |
| Goal setting | 1.1 Goal setting (behavior)/1.3 Goal setting (outcome) |
| Action planning | 1.4 Action planning |
| Performance feedback | 2.2 Feedback on behavior |
| Self-monitoring | 2.3 Self-monitoring of behavior/2.4 Self-monitoring of outcome(s) of behavior |
| Social support | 3.1-3 Items in group “Social support” |
| Rewards | 10.1-11 Items in group “Reward and threat” |
| Restructuring the physical environment | 12.1Restructuring the physical environment |
| Framing | 13.2 Framing/reframing |
|  |  |
