# Supplementary material for: Clinical Usability of Exercise Prescription Apps for Professional Use: Systematic Review and Multidimensional Evaluation
Source: JMIR Mhealth Uhealth. 2026 Mar 25;14:e77616. doi: 10.2196/77616 (PMC13015917; doi:10.2196/77616)
Supplement: Multimedia Appendix 3 [file mhealth-v14-e77616-s003.docx]

| Appendix 3: The Consensus on Exercise Reporting Template (CERT) checklist with detailed description among exercise prescription apps (n=6, 2024) | | | | | | | |
| --- | --- | --- | --- | --- | --- | --- | --- |
| Item description | | physiAPP | medbridge GO | Wibbi | Rehab Guru Client | trackactive pro-patient app | telehab |
|  | |  |  |  |  |  |  |
| **WHAT: materials** | |  |  |  |  |  |  |
| 1 | Detailed description of the type of exercise equipment (e.g. weights, exercise equipment such as machines, treadmill, bicycle ergometer etc) | 1 | 1 | 1 | 1 | 1 | 1 |
| **WHO: provider** | |  |  |  |  |  |  |
| 2 | Detailed description of the qualifications, teaching/supervising expertise, and/or training undertaken by the exercise instructor | 1 | 1 | 1 | 1 | 1 | 1 |
| **HOW: delivery** | |  |  |  |  |  |  |
| 3 | Describe whether exercises are performed individually or in a group | 1 | 1 | 1 | 1 | 1 | 1 |
| 4 | Describe whether exercises are supervised or unsupervised and how they are delivered | 1 | 1 | 1 | 1 | 1 | 1 |
| 5 | Detailed description of how adherence to exercise is measured and reported | 1 | 1 | 1 | 0 | 1 | 1 |
| 6 | Detailed description of motivation strategies | 0 | 0 | 0 | 0 | 0 | 0 |
| 7A | Detailed description of the decision rule(s) for determining exercise progression | 0 | 0 | 0 | 0 | 0 | 0 |
| 7B | Detailed description of how the exercise program was progressed | 0 | 0 | 0 | 0 | 0 | 0 |
| 8 | Detailed description of each exercise to enable replication (e.g. photographs, illustrations , video etc) | 1 | 1 | 1 | 1 | 1 | 1 |
| 9 | Detailed description of any home program component (e.g. other exercises, stretching etc) | 1 | 1 | 1 | 1 | 1 | 1 |
| 10 | Describe whether there are any non-exercise components (e.g. education, cognitive behavioural therapy, massage etc) | 1 | 1 | 1 | 0 | 0 | 1 |
| 11 | Describe the type and number of adverse events that occurred during exercise | 0 | 0 | 0 | 0 | 0 | 0 |
| **WHERE: location** | |  |  |  |  |  |  |
| 12 | Describe the setting in which the exercises are performed | 1 | 1 | 1 | 1 | 1 | 1 |
| **WHEN, HOW MUCH: dosage** | |  |  |  |  |  |  |
| 13 | Detailed description of the exercise intervention including, but not limited to, number of exercise repetitions/sets/sessions, session duration, intervention/program duration etc | 1 | 1 | 1 | 1 | 1 | 1 |
| **TAILORING:what, how** | |  |  |  |  |  |  |
| 14A | Describe whether the exercises are generic (one size fits all) or tailored whether tailored to the individual | 0 | 0 | 0 | 0 | 0 | 0 |
| 14B | Detailed description of how exercises are tailored to the individual | 0 | 0 | 0 | 0 | 0 | 0 |
| 15 | Describe the decision rule for determining the starting level at which people commence an exercise program (such as beginner, intermediate, advanced etc) | 0 | 0 | 0 | 0 | 0 | 0 |
| **HOW WELL: planned, actual** | |  |  |  |  |  |  |
| 16A | Describe how adherence or fidelity to the exercise intervention is assessed/measured | 1 | 1 | 1 | 1 | 1 | 1 |
| 16B | Describe the extent to which the intervention was delivered as planned | 0 | 0 | 0 | 0 | 0 | 0 |
| **Total score** | | 11 | 11 | 11 | 9 | 10 | 11 |
|  |  |  |  |  |  |  |  |
