# Supplementary material for: Clinical Usability of Exercise Prescription Apps for Professional Use: Systematic Review and Multidimensional Evaluation
Source: JMIR Mhealth Uhealth. 2026 Mar 25;14:e77616. doi: 10.2196/77616 (PMC13015917; doi:10.2196/77616)
Supplement: Multimedia Appendix 4 [file mhealth-v14-e77616-s004.docx]

| **Appendix 4: The behavior change technique(BCT) coded in Behavior Change Technique Taxonomy version 1 (BCTTv1) framework among exercise prescription apps (n=6, 2024)** | | | | | | | |
| --- | --- | --- | --- | --- | --- | --- | --- |
| **BCT Reference** | **BCT Name** | **physiAPP** | **medbridge GO** | **Wibbi** | **Rehab Guru Client** | **trackactive pro-patient app** | **telehab** |
| 1.1 | Goal setting (behavior) | 1 | 1 | 1 | 1 | 1 | 1 |
| 1.2 | Problem solving | 0 | 0 | 0 | 0 | 0 | 0 |
| 1.3 | Goal setting (outcome) | 1 | 0 | 0 | 0 | 0 | 1 |
| 1.4 | Action planning | 1 | 1 | 1 | 1 | 1 | 1 |
| 1.5 | Review behavior goal(s) | 1 | 1 | 1 | 1 | 1 | 1 |
| 1.6 | Discrepancy between current behavior and goal | 0 | 0 | 0 | 0 | 0 | 0 |
| 1.7 | Review outcome goal(s) | 1 | 0 | 0 | 0 | 0 | 1 |
| 1.8 | Behavioral contract | 0 | 0 | 0 | 0 | 0 | 0 |
| 1.9 | Commitment | 0 | 0 | 0 | 0 | 0 | 0 |
| 2.1 | Monitoring of behavior by others without feedback | 1 | 1 | 1 | 1 | 1 | 1 |
| 2.2 | Feedback on behavior | 1 | 1 | 1 | 1 | 1 | 1 |
| 2.3 | Self-monitoring of behavior | 1 | 1 | 1 | 1 | 1 | 1 |
| 2.4 | Self-monitoring of outcome(s) of behavior | 0 | 0 | 0 | 0 | 0 | 0 |
| 2.5 | Monitoring outcome(s) of behavior by others without feedback | 1 | 0 | 0 | 0 | 0 | 1 |
| 2.6 | Biofeedback | 0 | 0 | 0 | 0 | 0 | 0 |
| 2.7 | Feedback on outcome(s) of behavior | 0 | 0 | 0 | 0 | 0 | 0 |
| 3.1 | Social support (unspecified) | 0 | 0 | 0 | 0 | 0 | 0 |
| 3.2 | Social support (practical) | 0 | 0 | 0 | 0 | 0 | 0 |
| 3.3 | Social support (emotional) | 0 | 0 | 0 | 0 | 0 | 0 |
| 4.1 | Instruction on how to perform a behavior | 1 | 1 | 1 | 1 | 1 | 1 |
| 4.2 | Information about antecedents | 0 | 0 | 0 | 0 | 0 | 0 |
| 4.3 | Re-attribution | 0 | 0 | 0 | 0 | 0 | 0 |
| 4.4 | Behavioral experiments | 0 | 0 | 0 | 0 | 0 | 0 |
| 5.1 | Information about health consequences | 1 | 1 | 1 | 0 | 0 | 1 |
| 5.2 | Salience of consequences | 0 | 0 | 0 | 0 | 0 | 0 |
| 5.3 | Information about social and environmental consequences | 0 | 0 | 0 | 0 | 0 | 0 |
| 5.4 | Monitoring of emotional consequences | 0 | 0 | 0 | 0 | 0 | 0 |
| 5.5 | Anticipated regret | 0 | 0 | 0 | 0 | 0 | 0 |
| 5.6 | Information about emotional consequences | 0 | 0 | 0 | 0 | 0 | 0 |
| 6.1 | Demonstration of the behavior | 1 | 1 | 1 | 1 | 1 | 1 |
| 6.2 | Social comparison | 0 | 0 | 0 | 0 | 0 | 0 |
| 6.3 | Information about others' approval | 0 | 0 | 0 | 0 | 0 | 0 |
| 7.1 | Prompts/cues | 0 | 0 | 0 | 0 | 0 | 0 |
| 7.2 | Cue signalling reward | 0 | 0 | 0 | 0 | 0 | 0 |
| 7.3 | Reduce prompts/cues | 0 | 0 | 0 | 0 | 0 | 0 |
| 7.4 | Remove access to the reward | 0 | 0 | 0 | 0 | 0 | 0 |
| 7.5 | Remove aversive stimulus | 0 | 0 | 0 | 0 | 0 | 0 |
| 7.6 | Satiation | 0 | 0 | 0 | 0 | 0 | 0 |
| 7.7 | Exposure | 0 | 0 | 0 | 0 | 0 | 0 |
| 7.8 | Associative learning | 0 | 0 | 0 | 0 | 0 | 0 |
| 8.1 | Behavioral practice/ rehearsal | 1 | 1 | 1 | 1 | 1 | 1 |
| 8.2 | Behavior substitution | 0 | 0 | 0 | 0 | 0 | 0 |
| 8.3 | Habit formation | 0 | 0 | 0 | 0 | 0 | 0 |
| 8.4 | Habit reversal | 0 | 0 | 0 | 0 | 0 | 0 |
| 8.5 | Overcorrection | 0 | 0 | 0 | 0 | 0 | 0 |
| 8.6 | Generalisation of a target behavior | 0 | 0 | 0 | 0 | 0 | 0 |
| 8.7 | Graded tasks | 0 | 0 | 0 | 0 | 0 | 0 |
| 9.1 | Credible source | 1 | 1 | 1 | 1 | 1 | 1 |
| 9.2 | Pros and cons | 0 | 0 | 0 | 0 | 0 | 0 |
| 9.3 | Comparative imagining of future outcomes | 0 | 0 | 0 | 0 | 0 | 0 |
| 10.1 | Material incentive (behavior) | 0 | 0 | 0 | 0 | 0 | 0 |
| 10.2 | Material reward (behavior) | 0 | 0 | 0 | 0 | 0 | 0 |
| 10.3 | Non-specific reward | 0 | 0 | 0 | 0 | 0 | 0 |
| 10.4 | Social reward | 0 | 1 | 0 | 0 | 1 | 1 |
| 10.5 | Social incentive | 0 | 0 | 0 | 0 | 0 | 0 |
| 10.6 | Non-specific incentive | 0 | 0 | 0 | 0 | 0 | 0 |
| 10.7 | Self-incentive | 0 | 0 | 0 | 0 | 0 | 0 |
| 10.8 | Incentive (outcome) | 0 | 0 | 0 | 0 | 0 | 0 |
| 10.9 | Self-reward | 0 | 0 | 0 | 0 | 0 | 0 |
| 10.1 | Reward (outcome) | 0 | 0 | 0 | 0 | 0 | 0 |
| 10.11 | Future punishment | 0 | 0 | 0 | 0 | 0 | 0 |
| 11.1 | Pharmacological support | 0 | 0 | 0 | 0 | 0 | 0 |
| 11.2 | Reduce negative emotions | 0 | 0 | 0 | 0 | 0 | 0 |
| 11.3 | Conserving mental resources | 0 | 0 | 0 | 0 | 0 | 0 |
| 11.4 | Paradoxical instructions | 0 | 0 | 0 | 0 | 0 | 0 |
| 12.1 | Restructuring the physical environment | 0 | 0 | 0 | 0 | 0 | 0 |
| 12.2 | Restructuring the social environment | 0 | 0 | 0 | 0 | 0 | 0 |
| 12.3 | Avoidance/reducing exposure to cues for the behavior | 0 | 0 | 0 | 0 | 0 | 0 |
| 12.4 | Distraction | 0 | 0 | 0 | 0 | 0 | 0 |
| 12.5 | Adding objects to the environment | 0 | 0 | 0 | 0 | 0 | 0 |
| 12.6 | Body changes | 1 | 1 | 1 | 1 | 1 | 1 |
| 13.1 | Identification of self as role model | 0 | 0 | 0 | 0 | 0 | 0 |
| 13.2 | Framing/reframing | 0 | 0 | 0 | 0 | 0 | 0 |
| 13.3 | Incompatible beliefs | 0 | 0 | 0 | 0 | 0 | 0 |
| 13.4 | Valued self-identity | 0 | 0 | 0 | 0 | 0 | 0 |
| 13.5 | Identity associated with changed behavior | 0 | 0 | 0 | 0 | 0 | 0 |
| 14.1 | Behavior cost | 0 | 0 | 0 | 0 | 0 | 0 |
| 14.2 | Punishment | 0 | 0 | 0 | 0 | 0 | 0 |
| 14.3 | Remove reward | 0 | 0 | 0 | 0 | 0 | 0 |
| 14.4 | Reward approximation | 0 | 0 | 0 | 0 | 0 | 0 |
| 14.5 | Rewarding completion | 0 | 0 | 0 | 0 | 0 | 0 |
| 14.6 | Situation-specific reward | 0 | 0 | 0 | 0 | 0 | 0 |
| 14.7 | Reward incompatible behavior | 0 | 0 | 0 | 0 | 0 | 0 |
| 14.8 | Reward alternative behavior | 0 | 0 | 0 | 0 | 0 | 0 |
| 14.9 | Reduce reward frequency | 0 | 0 | 0 | 0 | 0 | 0 |
| 14.10 | Remove punishment | 0 | 0 | 0 | 0 | 0 | 0 |
| 15.1 | Verbal persuasion about capability | 0 | 0 | 0 | 0 | 0 | 0 |
| 15.2 | Mental rehearsal of successful performance | 0 | 0 | 0 | 0 | 0 | 0 |
| 15.3 | Focus on past success | 0 | 0 | 0 | 0 | 0 | 0 |
| 15.4 | Self-talk | 0 | 0 | 0 | 0 | 0 | 0 |
| 16.1 | Imaginary punishment | 0 | 0 | 0 | 0 | 0 | 0 |
| 16.2 | Imaginary reward | 0 | 0 | 0 | 0 | 0 | 0 |
| 16.3 | Vicarious consequences | 0 | 0 | 0 | 0 | 0 | 0 |
| 17.1 | Tailoring to demographic characteristics | 1 | 1 | 1 | 1 | 1 | 1 |
| 17.2 | Tailoring to health status | 1 | 1 | 1 | 1 | 1 | 1 |
| 17.3 | Tailoring to psychological characteristics | 0 | 0 | 0 | 0 | 0 | 0 |
| 17.4 | Adjusting intervention content to performance | 1 | 1 | 1 | 1 | 1 | 1 |
| 17.5 | General/Not Enough Detail | 0 | 0 | 0 | 0 | 0 | 0 |
| 18.1 | Earn points | 0 | 0 | 0 | 0 | 0 | 0 |
| 18.2 | Earn badges/levels | 0 | 0 | 0 | 0 | 0 | 0 |
| 18.3 | Leaderboards | 0 | 0 | 0 | 0 | 0 | 0 |
| 18.4 | Competitions | 0 | 0 | 0 | 0 | 0 | 0 |
| Total number of BCT with original version of BCTT v1(1.1-16.3) | | 15 | 13 | 12 | 11 | 12 | 16 |
| Total number of BCT with expanded version of BCTT v1(1.1-18.4) | | 18 | 16 | 15 | 14 | 15 | 19 |
|  |  |  |  |  |  |  |  |
