# Supplementary material for: Clinical Usability of Exercise Prescription Apps for Professional Use: Systematic Review and Multidimensional Evaluation
Source: JMIR Mhealth Uhealth. 2026 Mar 25;14:e77616. doi: 10.2196/77616 (PMC13015917; doi:10.2196/77616)
Supplement: Multimedia Appendix 5 [file mhealth-v14-e77616-s005.docx]

| Appendix 5: The Mobile App Rating Scale (MARS) scores respectively among exercise prescription apps on Android system(n=6, 2024) . | | | | | | | |
| --- | --- | --- | --- | --- | --- | --- | --- |
| Category | Item | physiAPP | medbridge GO | Wibbi | Rehab Guru Client | trackactive pro-patient app | telehab |
| Engagement | |  |  |  |  |  |  |
|  | Entertainment | 2 | 2 | 2 | 2 | 2 | 2 |
|  | Interest | 2 | 2 | 2 | 2 | 2 | 2 |
|  | Customisation | 3 | 3 | 1 | 2 | 1 | 2 |
|  | Interactivity | 3 | 3 | 2 | 2 | 2 | 3 |
|  | Target group | 4 | 4 | 4 | 4 | 4 | 4 |
| Functionality | |  |  |  |  |  |  |
|  | Performance | 5 | 4 | 5 | 4 | 5 | 4 |
|  | Ease of use | 5 | 4 | 5 | 5 | 5 | 5 |
|  | Navigation | 5 | 4 | 4 | 5 | 5 | 5 |
|  | Gestural design | 3 | 5 | 4 | 5 | 5 | 5 |
| Aesthetics | |  |  |  |  |  |  |
|  | Layout | 4 | 4 | 3 | 4 | 3 | 4 |
|  | Graphics | 5 | 5 | 5 | 5 | 5 | 5 |
|  | Visual appeal: | 3 | 3 | 2 | 3 | 2 | 3 |
| Information | |  |  |  |  |  |  |
|  | Accuracy of app description (in app store) | 5 | 4 | 4 | 4 | 4 | 5 |
|  | Goals | 5 | 4 | 4 | 4 | 4 | 5 |
|  | Quality of information | 5 | 5 | 5 | 5 | 5 | 5 |
|  | Quantity of information | 4 | 5 | 5 | 3 | 3 | 4 |
|  | Visual information | 4 | 4 | 4 | 4 | 4 | 4 |
|  | Credibility | 3 | 3 | 3 | 3 | 3 | 3 |
|  | Evidence base | 4 | N/A | N/A | N/A | N/A | 3 |
| Engagement Mean Score | | 2.80 | 2.80 | 2.20 | 2.40 | 2.20 | 2.60 |
| Functionality Mean Score | | 4.50 | 4.25 | 4.50 | 4.75 | 5.00 | 4.75 |
| Aesthetics Mean Score | | 4.00 | 4.00 | 3.33 | 4.00 | 3.33 | 4.00 |
| Information Mean Score | | 4.29 | 4.17 | 4.17 | 3.83 | 3.83 | 4.14 |
| App quality mean Score | | 3.90 | 3.80 | 3.55 | 3.75 | 3.59 | 3.87 |
|  |  |  |  |  |  |  |  |
